# The emerging roles of Gα12/13 proteins on the hallmarks of cancer in solid tumors

**DOI:** 10.1038/s41388-021-02069-w

**Published:** 2021-10-23

**Authors:** Suhail Ahmed Kabeer Rasheed, Lalitha Vaishnavi Subramanyan, Wei Kiang Lim, Udhaya Kumari Udayappan, Mei Wang, Patrick J. Casey

**Affiliations:** 1 Program in Cancer and Stem Cell Biology, Duke-NUS Medical School, Singapore, 169857 Singapore; 2 Dept. of Pharmacology and Cancer Biology, Duke Univ. Medical Center, Durham, NC 27710 USA

**Keywords:** Oncogenes, Cell signalling

## Abstract

G12 proteins comprise a subfamily of G-alpha subunits of heterotrimeric GTP-binding proteins (G proteins) that link specific cell surface G protein-coupled receptors (GPCRs) to downstream signaling molecules and play important roles in human physiology. The G12 subfamily contains two family members: Gα12 and Gα13 (encoded by the *GNA12* and *GNA13* genes, respectively) and, as with all G proteins, their activity is regulated by their ability to bind to guanine nucleotides. Increased expression of both Gα12 and Gα13, and their enhanced signaling, has been associated with tumorigenesis and tumor progression of multiple cancer types over the past decade. Despite these strong associations, Gα12/13 proteins are underappreciated in the field of cancer. As our understanding of G protein involvement in oncogenic signaling has evolved, it has become clear that Gα12/13 signaling is pleotropic and activates specific downstream effectors in different tumor types. Further, the expression of Gα12/13 proteins is regulated through a series of transcriptional and post-transcriptional mechanisms, several of which are frequently deregulated in cancer. With the ever-increasing understanding of tumorigenic processes driven by Gα12/13 proteins, it is becoming clear that targeting Gα12/13 signaling in a context-specific manner could provide a new strategy to improve therapeutic outcomes in a number of solid tumors. In this review, we detail how Gα12/13 proteins, which were first discovered as proto-oncogenes, are now known to drive several “classical” hallmarks, and also play important roles in the “emerging” hallmarks, of cancer.

## Introduction

G protein-coupled receptors (GPCRs) are the largest family of cell surface receptors, comprising nearly 1000 different members. GPCRs can be activated via several different types of ligands, ranging from photons to hormones and neurotransmitters [1, 2]. Because of the sheer number of GPCR-ligand combinations, these receptors play a major role in a number of important physiological functions of the human body, including hormonal signaling, neurotransmission, cardiac function, and cell growth. Deregulation of GPCR signaling can lead to numerous human diseases, including cancer [3]. As such, GPCRs are the most targeted receptor family, with over 30% of all drugs approved for various ailments being targeted at GPCRs [4, 5]. However, the understanding of the role of GPCRs in cancer biology is an emerging field, and the full scope of GPCR targeting in cancers has not yet been exploited. A recent ﻿Genomic Identification of Significant Targets in Cancer analysis of 28 different tumor databases from The Cancer Genome Atlas showed that nearly 424 different GPCRs and their associated heterotrimeric G proteins are dysregulated in cancer. In prostate and breast cancer alone, more than ten GPCRs have been implicated, with the most prominent associations seen for Cys-X-Cys Chemokine Receptor 4 (CXCR4), G protein-Coupled Receptor 19, Lysophosphatidic receptor 6, Protease Activated Receptor 1 and 2 (PAR-1 and -2), Prostaglandin E2 (PGE2) and Cholinergic Receptor, Muscarinic 1 [3]. Together, these findings highlight the potential of targeting GPCRs in cancer.

**Fig. 1:**
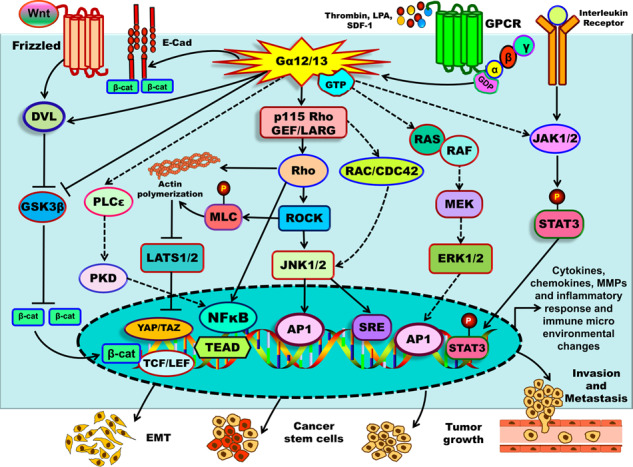
Signaling pathways regulated by Gα12/13 in solid tumors. Studies of the past decade have shown that Gα12/13 can potentially drive a number of signaling pathways that are implicated in tumorigenesis and metastasis. Some of the key pathways are shown in this figure, with signaling through Rho GTPases depicted in the central axis considered a dominant pathway for most of the biological consequences of Gα12/13 activation. See text for details and definition of acronyms.

GPCRs are coupled to heterotrimeric G proteins that are comprised of Gα, Gβ, and Gγ subunits. Based on sequence similarities, the Gα class can be further categorized into the Gs, Gq, Gi, and G12 subfamilies. All heterotrimeric G proteins function as molecular switches; ligand binding to the GPCR triggers a conformational change in the receptor, resulting in GDP-GTP exchange on the Gα subunit and its subsequent dissociation from the Gβγ dimer (Fig. 1). Both the GTP-bound Gα subunit and the Gβγ dimers each can engage a number of different downstream signaling pathways that ultimately result in specific cellular phenotypes or behaviors. The Gs family is known to activate adenylate cyclase and cAMP signaling, while the Gi family is known to inhibit the same signals. Gq family members control PLCγ, and hence calcium and PKC signaling pathways, while G12 family members (Gα12/13) are mostly described as acting via the small GTPase Rho [2, 6–8]. In terms of cancer signaling, all four families of G proteins Gs, Gq, Gi, and G12 proteins have been implicated in a variety of tumor types [6, 9–12]. In this review, we will focus on the role of Gα12/13 proteins in solid tumors.

As noted above, several GPCRs and their ligands have been implicated in tumorigenesis and metastasis. Most interestingly, many of these GPCRs signal through G12 proteins, and targeting these proteins could block the function of different GPCRs in various cancer cell types [13]. Both 43 kDa proteins, Gα12 and Gα13 are expressed in many tissues and are involved in multiple physiological and developmental processes [10, 14]. Mutations in Gα12/13 proteins in the Switch II region of the proteins in which a glutamine (Q) at position 231 is switched to Leucine (L) (Q231L) results in a GTPase-deficient and constitutively active protein [15]. Naturally occurring such activating mutations of Gα12/13 are seldom seen in solid tumors, with the exception of bladder carcinoma, where infrequent activating Gα13 mutations have been reported [16]. Instead, what is often observed is an increased expression of the wild-type forms of Gα12 and Gα13 in many solid tumors [3, 17, 18]. However, mutations in Gα13 (but not Gα12) have been recently described as the top five frequently occurring mutations in tumors of B-cell origins, including Burkitt’s and diffuse large cell B-cell lymphomas (DLBCL) [19–21]. Interestingly, the Gα13 mutations found in B-cell-derived lymphomas are loss-of-function mutations, suggesting a tumor suppressing role of Gα13 in these cell types [22]. While it is important to understand what is happening with Gα13 in B-cell lymphomas, in this review we will focus on the roles of Gα12/13 in oncogenesis and tumor progression with an emphasis on solid tumors.

The first report of a positive correlation between Gα12 protein expression and cancer aggressiveness came from immunohistochemical (IHC) analysis of tumor tissues showing that expression of Gα12 was enhanced during progression of breast and prostate cancers (Table 1) [17, 18]. A similar observation was later reported in Nasopharyngeal Carcinoma (NPC). The same study also showed that Gα12 protein expression correlated significantly with lymph node metastasis in NPC patients [23]. Subsequently, increased expression of Gα12 protein in Hepatocellular Carcinoma (HCC) was reported [24]. In an unbiased expression analysis of GNA12 mRNA expression across 32 different cancers, we found that eight tumors, including DLBCL, esophageal carcinoma, glioblastoma multiforme, head and neck squamous cell carcinoma (HNSCC), brain low-grade glioma, pancreatic adenocarcinoma, skin cutaneous melanoma, and thymoma, showed significant increases in GNA12 mRNA compared to their corresponding normal tissues, further confirming the implication of Gα12 in cancer (Table 1).

**Table 1:** Expression data for Gα12 and Gα13 in tumor tissues and cell lines, obtained either from (i) published reports, or (ii) database analysis (GEPIA, http://gepia.cancerpku.cn/index.html), as indicated.

Solid tumor types	Gα12-mRNA tumor vs normal	Gα12-protein tumor vs normal	Gα13-mRNA tumor vs normal	Gα13-protein tumor vs normal
Breast invasive carcinoma	No change Source: GEPIA	*High in metastatic tumors Source:* Kelly et al. [17]	*High in metastatic tumor cells* *Source:* Rasheed et al. [26]	*High in metastatic tumor cells Source:* Rasheed et al. [26]
Cervical carcinoma	**Low in tumors** **Source: GEPIA** ***P*** < 0.05	No report	No Change Source: GEPIA	No report
Cholangiocarcinoma	*High in tumors* *Source: GEPIA p* *<* *0.05*	No report	*High in tumors* *Source: GEPIA p* *<* *0.05*	No report
Esophageal carcinoma	*High in tumors* *Source: GEPIA p* *<* *0.05*	No report	*High in tumors* *Source: GEPIA p* *<* *0.05*	No report
Glioblastoma multiforme	*High in tumors* *Source: GEPIA p* *<* *0.05*	No report	*High in tumors* *Source: GEPIA p* *<* *0.05*	No report
Head and neck squamous cell carcinoma	*High in tumors* *Source: GEPIA p* *<* *0.05*	*High in tumors and metastases Source:* Liu et al. [23]	No Change Source: GEPIA	*High in tumors and metastases* *Source:* Rasheed et al. [28]
Lower grade glioma	*High in tumors* *Source: GEPIA p* *<* *0.05*	No report	*High in tumors* *Source: GEPIA p* *<* *0.05*	No report
hepatocellular carcinoma	No Change Source: GEPIA	*High in tumors and metastases Source:* Yang et al. [24]	No Change Source: GEPIA	No report
Ovarian serous cystadenocarcinoma	**Low in tumors** **Source: GEPIA** ***p*** < 0.05	*High in tumor cells* *Source:* Ha et al. [49, 50]	No Change Source: GEPIA	*High in tumor cells* *Source:* Cai and Xu [71]
Pancreatic adenocarcinoma	*High in tumors* *Source: GEPIA p* *<* *0.05*	No report	*High in tumors* *Source: GEPIA p* *<* *0.05*	No report
Prostate adenocarcinoma	No Change Source: GEPIA	*High in metastatic tumors* *Source:* Kelly et al*.* [17]	mRNA does not correlate to protein Source: Rasheed et al. [25]	*High in metastatic tumor cells* *Source:* Rasheed et al. [25]
Skin cutaneous melanoma	*High in tumors* *Source: GEPIA p* *<* *0.05*	No report	*High in tumors* *Source: GEPIA p* *<* *0.05*	No report
Stomach adenocarcinoma	*High in tumors* *Source: GEPIA p* *<* *0.05*	No report	*High in tumors* *Source: GEPIA p* *<* *0.05*	*High in tumors and metastases* *Source:* Zhang et al. [27]
Thymoma	*High in tumors* *Source: GEPIA p* *<* *0.05*	No report	No Change Source: GEPIA	No report
Uterine corpus endometrial carcinoma	**Low in tumors** **Source: GEPIA** ***p*** < 0.05	No report	No Change Source: GEPIA	No report

Values in italic reflect that the expression was higher in tumor cells/tissues compared to normal cells/tissues, while bold indicates reduced expression.

Similar to Gα12, Gα13 protein levels were also found to increase with the aggressiveness of breast and prostate cancer cells [25, 26]. In these studies, less invasive epithelial cells, such as LNCaP (prostate) and MCF-10A (breast), exhibited substantially lower expression of Gα13 compared to highly tumorigenic cells PC3 and DU145 (prostate) and MDA-MB-231 (breast) [25, 26]. In another study in two different cohorts of gastric cancer patients using IHC, high Gα13 expression was found in over 40% of the cases (Table 1). In addition, the same study showed that Gα13 protein expression was a biomarker for poor prognosis in gastric cancers [27]. Further, in a recent study, levels of Gα13 protein were identified as a biomarker for metastasis-free survival and drug resistance in HNSCCs. This study also reported an analysis of publicly available tumor gene expression databases showing that *GNA13* mRNA was upregulated in many solid tumors, and patients with higher *GNA13* expression showed significantly poorer overall survival in breast, lung, gastric, and ovarian cancers [28]. These data highlight the importance of amplified Gα12 and Gα13 expression in many different solid tumors, and suggest that these proteins could be potential prognostic biomarkers in a variety of solid tumors.

**Fig. 2:**
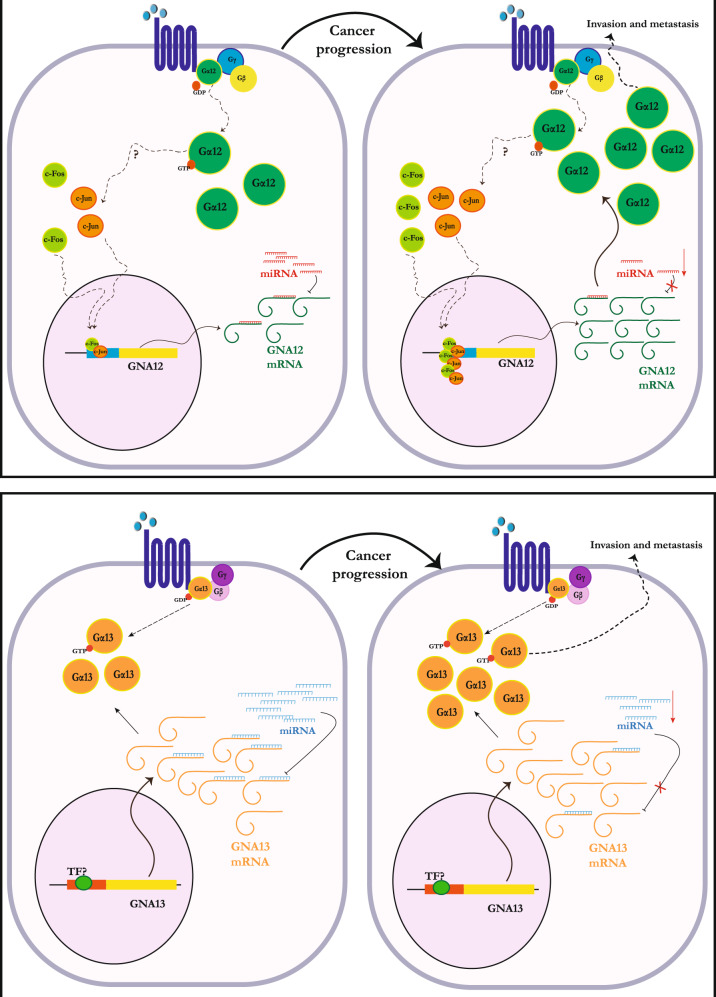
Mechanisms that drive increased expression of Gα12/13 proteins in cancers. **A** Expression of Gα12 is dysregulated mainly at the transcriptional level in a c-Jun/AP-1 dependent manner, at least in prostate cancer cells. Gα12 has also been shown to drive the activity of c-Jun in multiple solid tumors, suggesting the possibility of a feed forward loop. Dysregulation of Gα12 expression may also occur at a post transcriptional level, with the loss of at least one miRNA, miR-564 in breast cancer being shown to upregulate Gα12 expression. **B** Multiple studies indicate that expression of Gα13 is mainly regulated at the post-transcriptional level in solid tumors. Loss of several different miRNAs targeting GNA13 mRNA (miR-182, miR-200a, miR-31, miR-4731-5p, miR-30b-5p) during tumorigenesis and metastasis have been shown to lead to the increased Gα13 protein expression across multiple solid tumors. For both Gα12 and Gα13, the resultant overexpression of Gα12/13 then drives multiple steps of cancer progression. See text for details.

One of the important questions in the G12 field pertains to the mechanism of induction of Gα12/13 protein expression during progression of solid tumors. The observation that *GNA13* mRNA levels did not correlate with protein levels in a panel of prostate cancer cells led to the finding that Gα13 protein expression was post-transcriptionally regulated by microRNAs (miRNAs) miR-182 and miR-200a in a synergistic fashion. Mechanistically, binding of these miRNAs to the 3′-UTR of GNA13 did not alter mRNA expression but rather blocked translation of Gα13 protein in these cells [25]. Interestingly, a similar analysis in breast cancer cells showed that a different microRNA, miR-31, regulates expression of Gα13 in these cells [26]. Following these studies, a number of miRNAs were implicated in controlling Gα13 expression in different solid tumors, including miR-29c in colorectal cancers [29], miR-4731-5p in melanoma [30] and miR-30b-5p in renal cancers [31]. The expression of each of these miRNAs has been shown to be lost either via genomic deletions or via methylation during cancer progression, thus explaining the increase in Gα13 protein expression during tumor progression (Fig. 2) [25, 26, 29–31].

In contrast to the mechanisms that control Gα13 levels in cancers, both transcriptional and post-transcriptional mechanisms have been reported in control of Gα12 levels. In breast cancers, miR-564 was identified as one of the miRNAs that suppress Gα12 protein expression, as well as Gα12-induced tumorigenesis and metastasis [32]. In prostate cancer cells, however, Gα12 levels were shown to be primarily controlled at the transcriptional level [33]. In this study, c-Jun, an Activator protein-1 (AP-1) family transcription factor, was shown to bind directly to *GNA12* promoter and induce expression of Gα12. In this regard, it is important to note that Gα12 has been shown to induce c-Jun activity in cancer cells, so whether there is a feedforward loop that drives increased c-Jun-driven *GNA12* expression in these cells will be important to clarify. Another important point to note is that other mechanisms, such as genomic amplification and increased copy number, of *GNA12* and *GNA13*, have been recently reported [3]. Hence, it appears that several mechanisms, from genomic alterations to induction of transcription and post-transcriptional mechanisms, may drive expression of Gα12/13 proteins in various cells and tumors types.

**Fig. 3:**
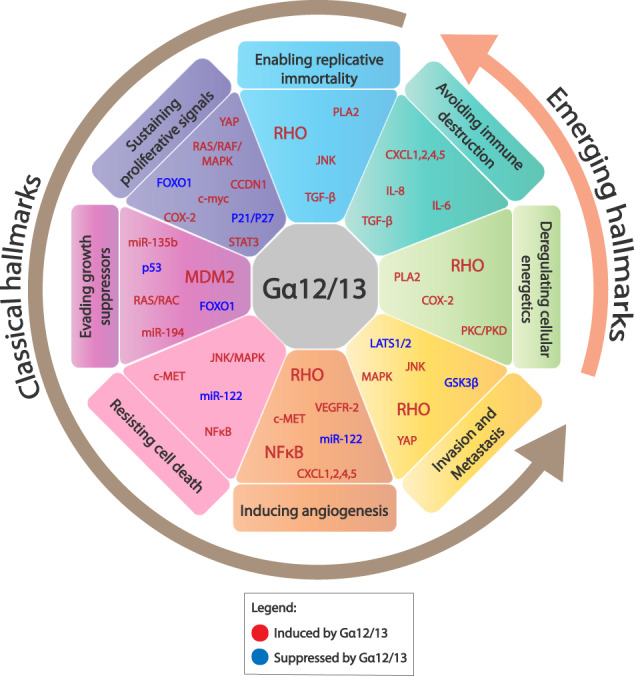
Schematic representation of the role of Gα12/13 in the hallmarks of cancer. The diverse roles of Gα12 and Gα13 allow their functions in cancer pathogenesis to be categorized according to the six ‘classical’ hallmarks and two ‘emerging’ hallmarks. Gα12/13 proteins impact the various hallmarks by either inducing oncogenic signaling pathways (labeled red) or suppressing tumor suppressive mechanisms (labeled blue). The degree of impact that Gα12/13 proteins have on each component may vary and this is reflected by the font size of the gene/pathway. See text for details.

For a very long time, cancer was considered primarily a disease characterized by uncontrolled cell division caused by genetic instability. However, it is now well-accepted that most human organs can, at various stages, harbor genetic mutations leading to hyper-proliferation of cells but do not manifest as a full-blown cancer. Several decades of research have led to the realization that the process of neoplastic transformation is a multistep process that exhibits several traits of a systemic disease. In 2000, Hanahan and Weinberg first detailed six recognizable traits that are commonly found in all types of solid and hematological cancers and termed them “Hallmarks of Cancer”. These are (i) enabling replicative immortality, (ii) sustaining proliferative signals, (iii) evading growth suppressors, (iv) resisting cell death, (v) inducing angiogenesis and (vi) invasion and metastasis (Fig. 3) [34]. A decade later, the same authors revisited these oncogenic traits based on new developments in the field and two additional ‘emerging’ hallmarks were added—(vii) deregulating cellular energetics and (viii) avoiding immune destruction—to the aforementioned “classical” hallmarks of cancer [35]. These guiding principles have changed the way in which the cancer field defines “oncogenes” and “tumor suppressors”. Previously, most oncogenes or tumor suppressors were believed to be drivers or suppressors, respectively, of only one hallmark, i.e., sustained cell proliferation. However, it is now appreciated that, based on several pieces of evidence, a qualified oncogene or a tumor suppressor can potentially impact several hallmarks of cancer. For example, the most commonly found oncogene, mutant RAS, and tumor suppressor, p53, have been linked to all hallmarks of cancer, including emerging hallmarks [36, 37]. Most importantly, studies in the past two decades have revealed that these hallmarks are intertwined in the larger process of tumorigenesis and tumor progression. GPCRs and heterotrimeric G proteins have been implicated in driving a number of these hallmarks of cancer, and a detailed review on this aspect has been recently published [12].

### Gα12/13-driven hallmarks of cancer

Both Gα12 and Gα13 were described as transforming oncogenes in the 1990s [38–40]. Research conducted on these two proteins in a range of solid tumors over the past three decades has implicated these proteins not only as important regulators of cell proliferation, but also as key drivers of several hallmarks of cancer. Here, we will provide an overview of the role of Gα12/13 proteins in driving the hallmarks of cancer, focusing on solid tumors (Fig. 3).

### Enabling replicative immortality

Cell replication is an essential process in the development and physiological functioning of multicellular organisms. However, in human biology, most cells undergo this cycle of cell growth and cell division a limited number of times. After a certain number of cell divisions, normal cells either enter an irreversible but viable state of nonproliferation termed cellular senescence, or undergo cell death. Interestingly, in cell culture experiments, it has been observed that some cells emerge with qualities of unlimited replication. This process is termed immortalization or transformation [34]. Work by Stuart Aaronson’s group on identification of potential oncogenes in soft tissue sarcoma identified Gα12 as a proto-oncogene. Transfection of wild-type Gα12 cDNA into NIH-3T3 fibroblast cells induced immortality and long-term growth in a soft agar assay [41]. A more potent ability to transform was observed with Gα12QL, the constitutively active mutant of Gα12 [42]. Soon after, wild-type Gα13 was also shown to transform NIH-3T3 cells [39]. These pioneering experiments led to discovery of two potential oncogenes, GNA12 and GNA13, and also showed that wild-type Gα12/13 proteins can be key drivers of enabling replicative immortality.

The mechanisms that mediate Gα12/13-induced replicative immortality are still being explored, but it was clear that this impact was serum-dependent. This study suggested that Gα12/13 proteins were likely not transformative on their own, but depended on factors present in serum, most likely ligands of GPCRs that couple to Gα12/13 [41]. Another report showed that Gα12-transformed NIH-3T3 cells not only formed colonies in soft agar in vitro but also formed tumors in mice in vivo. This study also reported that cells transformed with constitutively active Gα12 exhibited increased phosphorylation of phospholipase A2 (PLA2) together with increased secretion of arachidonic acid, and that blocking these components could inhibit Gα12-induced replicative immortality in these cells [42]. Additional studies further delineated the mechanism by which Gα12 induces transformation. Transformation of NIH-3T3 cells by Gα12 was found to be mediated by the activation of the small GTPase Rho and the serum response element (SRE)-induced transcriptional response [43]. In two other studies, Gα12-induced transformation of NIH-3T3 cells was shown to be amplified synergistically by co-transfection of c-raf-1 and an active mutant of Rac1 via activation of c-Jun N-terminal kinase (JNK) and c-fos-SRE-driven transcription [44, 45]. Of note, recently it was shown that this impact of Gα12/13-Rho signaling axis on transactivation of SRE is an evolutionarily conserved mechanism [46]. However, whether these findings reflected Gα12/13-induced replicative immortality was not clear at this stage, as most studies relied on overexpression of other players in the pathway. These reports did, however, show that wild-type forms of Gα12/13 are sufficient to enable replicative immortality in certain cells.

Gα12/13 have also been reported to be essential for expression of transforming growth factor β (TGF-β), and potentially for TGF-β-induced transformation and tumor progression in HCC. Thrombin, a ligand for the GPCR PAR-1 that couples to a number of G proteins including Gα12/13, was found to regulate expression of the TGF-β1 gene in a Gα12/13-dependent manner. This impact of Gα12/13-induced TGF-β1 mRNA expression was found to be dependent on activation of the Rho/Rac-mediated activation of AP-1 transcriptional program in liver cancer cells [40]. More recently, our own study showed that blocking expression of Gα13 alone is sufficient to suppress tumor initiation in primary head-and-neck cancer cell lines in mouse xenograft experiments [28]. These studies provide strong evidence that blocking Gα12/13 or their downstream effectors could block replicative immortality and abrogate tumor growth in many solid tumors (Figs. 1 and 3). It is important to note here that replicative immortality is considered to be mainly driven by aberration in telomere maintenance of cancer cells [34], and whether Gα12/13 driven signaling plays a role in this aspect is not yet known.

### Sustaining proliferative signals

As mentioned earlier, the most fundamental characteristic of a cancer cell is its ability to sustain proliferation. In normal tissues, cells proliferate upon receiving external stimuli from the environment, usually a growth factor that induces a signaling cascade that leads to cell proliferation. In cancer, cells can acquire the ability to proliferate independent of growth factors in a number of different ways. These mechanisms include either acquiring oncogenic mutations or increasing expression of signaling components that act downstream of growth-inducing cell surface receptors; the latter mechanism appears to be the one by which Gα12/13 proteins impact cell proliferation. However, although not quite physiological, studies with constitutively active Gα12/13 are common and do provide insight into processes potentially impacted by these proteins. In this regard, Gα12QL expression induced the metabolism of prostaglandins via increasing both secretion of arachidonic acid and expression of cyclooxygenase 2 (COX2) enzyme, a key mediator of prostaglandin synthesis. More importantly, this study showed that blocking COX2 or arachidonic acid suppressed DNA synthesis, thus blocking cell proliferation [47]. In NIH-3T3 cells, Gα12QL-driven proliferation was shown to be dependent on the STAT3 transcription factor via platelet-derived growth factor receptor α as well as activation of a non-receptor kinase Janus kinase 3 [15]. Importantly, in this same model system, another group showed that wild-type Gα12 (but not Gα13) mediates lysophosphatidic acid (LPA, a ligand for a G12-coupled GPCR)-driven DNA synthesis and cell proliferation [48]. In a follow-up study in a different cancer cell model, the LPA-LPAR axis was shown to be a key mediator of ovarian cancer cell proliferation. Notably, this study showed that Gα12-dependent mitogenic signaling by LPA involves activation of the cAMP-response element binding protein-induced transcriptional program [49]. A similar effect was observed in another ovarian cancer cell line, SKOV3, where silencing of Gα12 suppressed serum/LPA-induced cell proliferation in vitro and blocked tumor growth in vivo [50].

In lung cancer, silencing of Gα12 and Gα13 expression blocked cell proliferation in the small cell lung cancer (SCLC) cell line, H510. Gα12 was also found to have a more dominant effect on cell proliferation than Gα13 in these cells. However, no impact was observed with a cell line that was derived from a non-small cell lung cancer (NSCLC), A549, suggesting that the impact of Gα12/13 proteins is tumor subtype-specific in lung cancers. Indeed, a cell lineage-specific impact of Gα12/13 proteins has been consistently observed in multiple solid tumors [3, 8, 51]. Recent investigations using a synthetic biology approach in which artificial ligands were used to stimulate GPCR-Gα12/13 signaling found that Gα12/13-induced proliferation of ovarian cancer cells is mediated via the activation of the transcriptional coactivator YAP1, a key component of the Hippo signaling pathway [52]. In gastric cancer, ectopic expression of wild-type Gα13 promoted cell cycle progression and cell proliferation in vitro and also tumor growth in vivo by activating AKT and ERK1/2 signaling, c-myc activation and induction of cyclin-dependent kinase (CDK) regulator cyclin D1 expression. On the other hand, Gα13 expression also suppressed cell cycle inhibitors such as Forkhead Box O1 (FOXO1) transcription factor and the CDK inhibitors p21 and p27 [27]. These studies have provided considerable evidence of a role of Gα12/13 proteins in driving sustained cell proliferation, albeit in a context-specific manner. Interestingly, these studies also indicated that Gα12/13 proteins, in addition to their ability to interact with GPCRs and active Rho GTPases, could potentially cooperate with receptor and non-receptor kinases and activate multiple oncogenic signaling pathways.

### Evading growth suppressors

Along with acquiring the capabilities of replicative immortality and sustaining cell proliferation, cancer cells must also be able to evade robust mechanisms that suppress cell proliferation. The most frequently mutated tumor suppressor gene is TP53 (encoding the p53 protein), which is a transcription factor that becomes activated upon DNA damage and induces a DNA damage response. Loss-of-function mutations of p53 lead to tumorigenesis and metastasis and are found in nearly 50% of all tumors. The first study linking Gα12/13 and p53 was in MCF-10A, a non-tumorigenic breast epithelial cell line, where enforced expression of Gα12QL and Gα13QL induced expression of matrix metalloproteinase 2 (MMP-2) by promoting binding of p53 to the MMP-2 promoter. Moreover, blocking p53 suppressed Gα12/13QL-induced expression of MMP-2 and cell invasion and migration, indicating a tumor-promoting role for p53 rather than a tumor suppressive one in this context [53]. Separately, examination of HCC cell lines and tumor tissues showed increased expression of Gα12 and loss of FOXO1 protein, a transcription factor known for its role as a tumor suppressor. Further, enforced expression of Gα12/13QL suppressed FOXO1 expression in HCC cells, and the opposite was seen upon knockdown of endogenous Gα12. This Gα12/13-mediated inhibition of the FOXO1 tumor suppressor led to increased cell proliferation and tumor growth of HCC cells [54]. Consistent with these findings, another study in HCC cells showed that Gα12 suppressed expression of p53 protein by increasing production of MDM2 through the AP-1 signaling pathway. Gα12-induced suppression of p53 in these cells promoted cancer cell invasion, migration and tumor growth in vivo through induction of the proto-oncogene ZEB1, which is regulated via p53-responsive miRNAs [55]. These data highlight important but still emerging roles of Gα12/13 signaling in regulating tumor suppressive pathways.

### Resisting cell death

A major reason why tumors are able to grow unrestricted is due to the ability of cancer cells to develop resistance to apoptosis, or programmed cell death. Resistance to apoptosis is one of the classical hallmarks that is found in all types of cancers, and contributes to tumorigenesis, tumor progression and most importantly, resistance to treatment, as many anticancer therapies work primarily by activating apoptosis [56].

G12 proteins have been implicated in both the induction and suppression of apoptosis in a tumor- and cell-type-specific manner. Early reports on CHO-derived epithelial cells and COS-7 fibroblast cells indicated that enforced expression of Gα13QL induced apoptosis even in the presence of serum-containing media [57]. However, in mouse embryonal teratocarcinoma F9 cells, expression of either Gα12QL or Gα13QL promoted resistance to retinoic acid-induced apoptosis, and antisense RNA targeting of either protein rendered the cells more sensitive to retinoic acid-induced cell death [58]. In the same cell type, moesin knockdown resulted in a form of apoptosis termed anoikis, and expression of Gα13QL in moesin-depleted cells protected them from this process [59, 60]. In another study, a screen for biomarkers for gemcitabine resistance identified GNA13 as one of the genes that is highly correlated with resistance to gemcitabine-induced cell death in NSCLC cells [61]. Notably, endogenous Gα13 and RhoA expression were shown to drive cell fusion and resistance to 5-fluorouracil- and/or oxaliplatin-induced apoptosis in colorectal cancer cells [62].

In melanoma cells, the cytomegalovirus-induced GPCR US28 promoted cell death, and silencing Gα13 suppressed US28-driven apoptosis in these cells [63]. In HCC cell lines Huh7 and HepG2, a screen for Gα12-driven microRNAs identified miR-122 as one of the miRNAs that was significantly downregulated upon overexpression of Gα12QL, and knockdown of endogenous Gα12 upregulated expression of miR-122 in both lines. This Gα12-induced downregulation of miR-122 increased expression of its target c-MET, which blocked apoptosis and induced tumor growth [24]. In a recent study, our group reported that Gα13 (but not Gα12) is a biomarker for drug resistance in HNSCCs. Using a panel of primary HNSCC cells, high Gα13-expressing cells were found to be more resistant to apoptosis induced by multiple chemo- and radio-therapies, including cisplatin, the first-line therapy in HNSCC management. Similarly, enforced expression of Gα13 in HNSCC cells with low levels of the protein rendered them more resistant to cisplatin-induced apoptosis, and cells expressing high Gα13 became more sensitive to cisplatin-mediated cell death treatment upon knockdown of Gα13. Using a signaling pathway reporter screen, mitogen-activated protein kinase kinase (MAP2K or MEK1) and NF-κB signaling pathways were identified as key mediators of this Gα13-induced multidrug resistance [28]. These data revealed that elevated levels of Gα12/13 can be suppressors of apoptosis in cancer cells, and that targeting these proteins is a potential strategy to improve therapeutic outcomes in solid tumors.

### Inducing angiogenesis

Like normal tissues, the growth of a tumor requires nutrients and oxygen supply for its survival. During development and other physiological processes such as wound healing, this need is fulfilled by the formation of new blood vessels, a process termed angiogenesis. Tumor cells cleverly hijack these physiological mechanisms to induce tumor angiogenesis, and this process is important not only for survival of tumor cells but also for their ability to metastasize to distant organs [64]. A role for Gα13 in angiogenesis was revealed from knockout of both copies of the gene in mice, where embryonic lethality was found to be due to lack of blood vessel formation [11, 65].

The vascular endothelial growth factors VEGF1 and 2, and their putative receptors (VEGFR1 and 2) are key activators of both physiological and tumor angiogenesis. VEGFR2 gene expression was found to be markedly reduced in endothelial cells lacking Gα13. In mice with endothelial cell-specific knockout of Gα13, xenograft growth of Lewis lung carcinoma tumor was significantly reduced as was tumor angiogenesis [66]. In another study, enforced expression of Gα12QL in Huh7 cells led to the activation of c-Met, resulting in tumor growth and metastasis in mouse models. Interestingly, conditioned media from Gα12QL-expressing Huh7 cells promoted capillary tube formation of bovine aortic endothelial cells, indicating that Gα12 signaling in tumor cells leads to the secretion of certain pro-angiogenic factors [24]. Other reports have indicated that expression of several CXC family chemokines that function as potent endothelial cell chemoattractants and promoters of angiogenesis is increased by enforced expression of Gα13 in colorectal cancer cells. Silencing Gα13 in the same cells suppressed tumor growth and angiogenesis in vivo [67]. A similar observation has been made in prostate cancer cells, in which Gα13 was identified as a driver of the pro-angiogenic chemokine, CXCL5 [68]. In certain tumor types, the impact of Gα13 levels on CXC-chemokine expression appears to be mediated via Rho-induced transactivation of NF-κB [67, 68]. These studies indicate that elevated expression of Gα12/13 in tumor cells can potentially alter the tumor microenvironment by modulating secretion of several chemokines that promote endothelial cell recruitment and tumor angiogenesis, ultimately aiding tumor growth.

### Invasion and metastasis

Among all six classical hallmarks of cancer, invasion and metastasis are the most deleterious in terms of clinical outcome [69]. Tumor metastasis is a multistep process that involves the migration and invasion of tumor cells into the local microenvironment, followed by their intravasation across blood vessels and spread through the circulation to distant organs. This is followed by extravasation from the vessel at the metastatic site, and finally formation of micrometastases and the ultimate growth of a macrometastatic tumor [35]. While it is not possible to review here all the studies conducted in the past two decades that highlighted the importance of Gα12/13 proteins in metastasis, this topic has been reviewed previously [10, 70]. We will limit our focus here on recent studies that advanced our understanding of the roles and mechanisms of Gα12/13-driven cancer cell invasion and metastasis.

The first studies linking Gα12/13 proteins to tumor progression were performed in breast and prostate cancers, where increased levels of these proteins correlated with cancer progression. In these studies, blockade of Gα12/13 signaling using a specific dominant-negative construct, p115-RGS, suppressed Gα12QL-driven cancer cell invasion, and distant metastasis in an orthotopic mice model of breast and prostate cancers [17, 18]. Recently, we also showed that blocking endogenous expression of Gα13 alone using specific miRNAs significantly suppressed the migration and invasion of cancer cells [25, 26]. Several other groups have shown similar impact of Gα12 and Gα13 in inducing cancer cell migration and invasion in a number of cancer cell types, including ovarian, gastric, liver, pancreatic, colorectal, and head and neck cancers [24, 27–29, 31, 71–73]. Further, Stromal Derived Factor 1-induced CXCR4-Gα13-Rho signaling axis was shown to be important not only for cancer cell invasion and local migration but also for transendothelial migration, an important step for breast cancer cell intravasation into blood vessels and extravasation into a distant site of metastasis [74].

For Gα12/13-induced migration and invasion, the most common mechanism of action reported in several solid tumors is the activation of Rho GTPase-induced actin cytoskeleton modification and induction of the JNK signaling pathway [70, 71, 75–78]. Gα12/13-induced activation of the small GTPases Rho and in few instances Rac is a well-established mechanism that is mediated via specific Rho guanine nucleotide exchange factor (GEF) proteins, mainly p115-RhoGEF, leukemia-associated RhoGEF and PDZ-RhoGEF [79]. In ovarian cancer cells, a non-canonical GEF protein termed Ric-8A was shown to bind directly to Gα13 and potentiate Rho activity, leading to increased cancer cell migration and invasion. Interestingly, this Ric-8A mediated potentiation of Gα13 function appears independent of the canonical GPCR-mediated signaling [80]. In contrast, in one study in melanomas, ﻿Gα13 was shown to inhibit cell invasion through p190RhoGAP-mediated suppression of Rho activity [81]. Another interesting study reported that CD97, an adhesion GPCR, heterodimerizes with LPAR1 leading to Gα12/13-Rho-mediated prostate cancer cell invasion and bone metastasis [82]. Further, while many studies have shown the importance of the Rho signaling axis in Gα12/13-driven cancer cell migration and invasion, Rho-independent pathways have also been implicated in these phenotypes. In HeLa cells, Gα13-induced migration was reported to involve a direct interaction of Gα13 with JNK-associated leucine zipper protein to activate MAPK signaling directly, thus bypassing the requirement of Rho GTPases (Figs. 1 and 3) [83].

Recently, additional mechanisms, both Rho-dependent and Rho-independent, for Gα12/13-mediated invasion and metastasis have been discovered. The Wnt signaling pathway is implicated in cancer cell invasion and metastasis in many different solid tumors. Wnts signal through the frizzled (FZD) family of heptahelical receptor in the GPCR superfamily and activate β-catenin, a transcriptional coactivator of TCF/LEF transcription factor-driven gene expression program. Along with FZD, the low-density lipoprotein receptor-related proteins 5 and 6 function as co-receptors to activate Wnt-β-catenin signaling, while the orphan tyrosine kinase receptors ROR1/2 can also function as FZD co-receptors that trigger alternative Wnt signaling pathways. Recently, ROR1/2 was shown to suppress the phosphorylation of large tumor suppressor kinase 1/2 (LATS1/2) by activating the Gα12/13-Rho signaling axis, leading to YAP activation and subsequent stimulation of an alternative Wnt signaling pathway and resulting in cancer cell migration [84]. This is consistent with a report which found that LPA and sphingosine-1-phosphophate act through Gα12/13-coupled receptors to inhibit Lats1/2 [85]. In another study in ovarian cancer cells, LPA-induced migration was shown to be mediated via activation of Rho and Rho-associated kinase (ROCK) followed by dephosphorylation of YAP, leading to YAP activation. Crucially, this dephosphorylation was reported to be mediated by Gα13-mediated induction of protein phosphatase 1 [71]. In this regard, a similar impact was reported in another study, also in ovarian cancer, where Gα13 was shown to drive YAP activation and promote cancer cell migration by inducing ubiquitin-mediated degradation of LATS1, a repressor of YAP1 [86]. In colorectal cancers, loss of miR-29C resulted in increased expression of Gα13 and PTP4A1 and promoted invasion and metastasis. In this study, Gα13 was shown to phosphorylate and activate AKT, which in turn phosphorylated glycogen synthase kinase 3 beta (GSK3β). This Gα13-AKT-mediated inhibition of GSK3β activity led to increased β-catenin translocation to the nucleus and induced a transcriptional program that promoted cancer cell invasion and metastasis [29]. A separate study in colorectal cancer showed that PAR1 mediates β-catenin stabilization independently of the Wnt signaling machinery. This study proposed a novel cascade of the PAR1-induced Gα13-disheveled (DVL) axis in β-catenin stabilization and cancer cell invasion and metastasis [87]. In addition to these, our own studies demonstrated that Gα12/13 interact directly with the cytoplasmic domain of E-cadherin (E-cad) and cause the release of the transcriptional activator β-catenin, leading to loss of cell–cell adhesion, which is an initial step of the epithelial to mesenchymal transition (EMT) and cancer cell migration [88, 89].

Estrogen hormone binding to either of its receptors, estrogen receptor α and β (ERα and β), through extranuclear signaling can drive actin cytoskeleton modification, cancer cell migration, invasion, and metastasis via phosphorylation of moesin in ER-positive breast cancer cells [90]. In one study, Gα13 was reported to interact directly with ERα and this interaction led to activation of Rho- and ROCK2-mediated phosphorylation of moesin, thereby facilitating migration, invasion and metastasis in breast cancers. Similar findings were also observed with another female reproductive hormone, progesterone [90, 91]. Altogether, these studies indicate that Gα12/13 proteins drive several steps of the cancer cell invasion and metastasis cascade through classical Rho-mediated signaling pathways and via crosstalk with several other pro-metastatic signaling pathways such as Wnt/β-catenin, Hippo-YAP, tyrosine kinases, and sex hormones.

### Deregulating cellular energetics

A direct link between Gα12 signaling and deregulation of energy metabolism in cancer cells was observed several years ago when, as noted above, Gα12QL-transformed NIH-3T3 cells showed increased secretion of arachidonic acid [47]. A similar impact was reported for Gα13 in CHO cells, where enforced expression of Gα13QL, or activation of wild-type Gα13 via thrombin stimulation, resulted in PLA2 phosphorylation and increased prostaglandin production. The impact of Gα13 on PLA2 phosphorylation was shown to be mediated through activation of RhoA and consequently ERK1/2 signaling [92]. Activation of PLA2 and increased secretion of prostaglandins have been often linked to tumorigenesis and metastasis, and targeting such pathways has been proposed as a potential therapeutic strategy for treating solid tumors [93, 94].

LPA is another key product of fatty acid metabolism that has been implicated in several aspects of tumor growth and progression. Both PGE2 and LPA can be secreted into the surrounding microenvironment and stimulate cell proliferation through both autocrine and paracrine mechanisms [93]. LPA is a ligand of several LPA receptor family members (LPARs), which are frequently upregulated in solid tumors. More importantly, in the context of this review, these receptors are known to signal through Gα12/13 [4, 48, 95–98]. Another key signaling pathway implicated in cellular energetics in cancer cells is protein kinases C and D (PKC/PKD) signaling pathway [99–101]. Both Gα12 and Gα13, through Rho GTPases, have been shown to induce PKC and PKD signaling pathways, leading to increased tumorigenesis and metastasis [102, 103]. However, whether Gα12/13 signaling is indeed central to deregulation of cancer metabolic pathways is still an emerging field of study.

### Avoiding immune destruction

During the course of tumor development, host immune cells such as macrophages, natural killer cells of the innate arm, and cytotoxic T CD8^+^ and CD4^+^ cells of the adaptive arm of the immune system recognize antigens expressed on tumor cells and eliminate them, thus preventing development of a tumor. Cancer cells that overcome this immune cell-mediated killing are those that form tumors and progress [3, 35, 104]. Cancer cells have adopted two distinct mechanism to achieve this immune evasion. The first is direct inhibition of cytotoxic T cell activation by expressing cell surface programmed cell death ligand-1, which binds to its cognate receptor, programmed death protein-1 on cytotoxic T cells to suppress activation of these effector T cells. The second mechanism involves induction of the secretion of a plethora of cytokines and chemokines, such as IL-6, IL-8, TGF-β, and other CXC chemokines that recruit immune suppressive cells, such as M2 macrophages, CD4^+^ T regulatory (T_reg_) cells, and myeloid-derived suppressive cells (MDSCs) [105, 106]. Therefore, targeting cell intrinsic mechanisms that drive secretion of these chemokines could cause these tumors to become more responsive to immune cell-mediated tumor killing and be more sensitive to immune checkpoint blockade therapies.

Recently, we and others found that Gα12, but not Gα13, has a distinct function in stimulating the production of IL-6 and IL-8 [77, 107]. Both IL-6 and IL-8 are cytokines that mediate their activity through specific receptors; those for IL-8, IL-8RA (CXCR1), and IL-8RB (CXCR2), are GPCRs, whereas the receptor for IL-6, IL6R, transmits signals through gp130 and JAK-STAT signaling [108, 109]. Despite differences in their signaling processes, IL-6 and IL-8 have recently been implicated in modulating the tumor microenvironment by regulating the function and activity of tumor-associated immune cells [108, 109]. In another study, Gα12/13 signaling was shown to induce TGF-β, another family of immune-suppressive and tumor-promoting cytokines [40]. TGF-β is a potent immune suppressive cytokine in several cancers and impacts immune response by inhibiting growth and function of CD8^+^ and CD4^+^ T cells, B cells and also the innate immune cell types. In addition, TGF-β signaling expands the immune suppressive T_reg_ cell population within the tumor microenvironment, making it more immunologically quiescent and thus aiding the growth of the tumor [110]. More recently, work on colorectal and prostate cancers showed that Gα13, through Rho-mediated transactivation of NF-κB, induces a set of CXC family chemokines [67, 68]. These CXC family chemokines are known to bind to their receptors CXCR1 and CXCR2, which are highly expressed in several immune cell populations, particularly those with immune suppressive capabilities such as MDSCs and T_reg_ cells [111, 112]. For example, increased secretion of CXCL5 in prostate cancers is known to recruit MDSCs and T_reg_ cells to the tumor microenvironment, which blocks cytotoxic T cell activity and protects tumor cells from T cell-mediated cell death [113]. Therefore, Gα12/13 signaling potentially contributes to regulation of the tumor microenvironment in a unique manner by inducing an immune-evading phenotype. It is now important to delineate the mechanisms that drive Gα12/13-induced cytokine production and further investigate the capacity of Gα12/13 in regulating cancer immune phenotypes.

## Gα12/13-driven cancer stemness as a potential mechanism of oncogenesis and metastasis in solid tumors

**Fig. 4:**
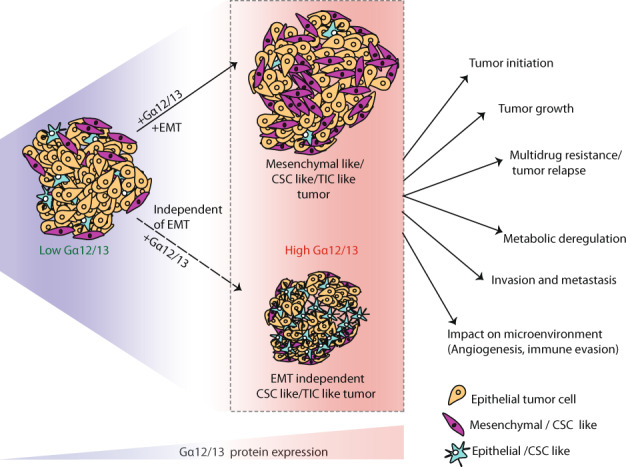
Gα12/13-promoted stemness is a potential mechanism of cancer progression, metastasis and drug resistance in tumors. Gα12/13 has been shown to drive both EMT-dependent and -independent mechanisms of cancer stem cells/tumor-initiating cells (CSCs/TICs) formations. Both proteins have also been reported to drive EMT and stemness in cancers through multiple signaling mechanisms. CSC-like phenotype induced by Gα12/13 can then contribute to multiple steps in cancer initiation and progression as shown in the figure. See text for details.

Historically, tumors were considered a homogenous group of cancer cells with similar genetic and phenotypic properties. However, it is now clear that a tumor, similar to a human organ, is a heterogeneous mix of cancer cells coexisting with a variety of immune cells, endothelial cells, pericytes, cancer-associated fibroblasts, etc., [35]. Importantly, in solid tumors, most tumor cells in the early stages of carcinomas are in fully differentiated, epithelial-like states. As the tumor progresses, a few cells de-differentiate into a mesenchymal-like phenotype in a process termed the EMT [114]. These few mesenchymal-type cells are also commonly described as cancer stem cells (CSCs) or tumor-initiating cells (TICs) due to their similarities with stem cells [115]. These CSC-like cells have been implicated in the entire process of cancer pathogenesis [116], including tumor initiation and growth [117], apoptosis resistance [118, 119], angiogenesis [120–123], invasion and metastasis [124, 125], immune evasion [126], and deregulated cellular energetics (Fig. 4) [127]. Based on these revelations, signaling pathways that mediate EMT/CSC induction in tumors have become some of the most sought-after targets for cancer therapy. Since Gα12/13 is able to mediate several steps of the process of tumorigenesis, drug resistance and metastasis, it appears that the ability of Gα12/13 signaling to induce EMT/CSC-like phenotype might be a common mechanism of the pleiotropic impact of elevated Gα12/13 signaling on the biology of solid tumors.

A role for Gα12/13 proteins in driving EMT/CSC-like phenotypes in solid tumors has emerged from several lines of investigation. As noted above, Gα13 signaling, through activation of AKT-GSK3β signaling, led to the loss of epithelial cell marker E-cad, gain of the mesenchymal cell marker vimentin and an increase in cancer cell invasion and metastasis [29]. Additionally, in HCC cells, enforced expression of Gα12QL induced EMT, cancer cell invasion, tumor growth, and metastasis in mice, while knockdown of wild-type Gα12 significantly blocked these processes. In this study, enforced expression of Gα12-induced EMT by activating ZEB1 transcriptional program via suppression of the p53-responsive microRNAs mir-200a/b, −192 and −215 [55]. A similar impact was reported for another microRNA, miR-564, in breast cancer cells. Here, inhibition of miR-564 induced the expression of Gα12, which partially, and in conjunction with PI3K-AKT signaling, induced the loss of epithelial markers E-cad and Zonula Occludens 1 (ZO-1) and gain of several mesenchymal markers such as ﻿fibronectin, SNAI2, ZEB1, and ZEB2, thereby indicating a strong EMT phenotype [32]. Consistent with this, our lab has observed that enforced expression of Gα13 in breast epithelial cells induced TGF-β signaling and an EMT-like gene expression signature [128]. In another study on renal cancer, loss of miR-30-5p was shown to induce EMT, growth, and metastasis by inducing expression of Gα13 [31]. Gα13 signaling was shown to induce cell proliferation, migration, invasion, and metastasis via activation of the EMT program in ovarian cancer cells [74]. In this regard, we found that Gα13-induced multidrug resistance in primary HNSCC cells through induction of a CSC/TIC-like phenotype. Enforced expression of Gα13 in HNSCC cells expressing relatively low levels of Gα13 protein promoted CSC marker expression and long-term spheroid growth in serial re-plating assays in vitro. Most importantly, in a serial dilution xenograft experiment, cells expressing high levels Gα13 initiated tumors much earlier, and in much lower cell numbers, than cells in which Gα13 expression had been silenced. [28]. Together, these studies show that Gα12/13 signaling can impact several hallmarks of cancer through induction of a common tumorigenic program involving an EMT/CSC-like transition (Fig. 4).

## Conclusion: perspectives on targeting G12 proteins as cancer therapy

It is now abundantly clear that GPCR signaling components, including Gα12/13 proteins, are involved in multiple hallmarks of cancers [12]. However, attempts at targeting individual GPCRs as a therapeutic strategy in cancer, such as using small molecule inhibitors of CXCR4 in solid tumors, have not yet demonstrated major benefit in the clinic [129]. A more effective approach may be to simultaneously target several GPCRs, or target common effectors that mediate signaling through various tumor-associated GPCRs. Interestingly, many GPCRs that have been implicated in tumorigenesis and tumor progression in solid tumors rely at least in part on Gα12/13 for downstream signaling. In this regard, genetic suppression strategies have provided compelling evidence for therapeutic benefit in targeting Gα12 and/or Gα13 in particular cancers. As an intracellular signaling protein with defined partners and potentially druggable pockets, it is certainly conceivable that one may also be able to identify small molecules targeting these proteins. Similar efforts in direct targeting of another family of heterotrimeric G protein, Gαq/11/14 which are structurally similar to Gα12/13, using naturally derived small molecule inhibitors have shown success in the pre-clinical settings [130]. Whichever route proves most tractable, there is clearly a need to develop Gα12/13-specific inhibitors to evaluate in treatment of cancers, and potentially other diseases where increased expression and/or activity of Gα12/13 has been implicated in disease progression [14].
